# Hollow Particles Obtained by Prilling and Supercritical Drying as a Potential Conformable Dressing for Chronic Wounds

**DOI:** 10.3390/gels9060492

**Published:** 2023-06-16

**Authors:** Maria Rosaria Sellitto, Chiara Amante, Rita Patrizia Aquino, Paola Russo, Rosalía Rodríguez-Dorado, Monica Neagu, Carlos A. García-González, Renata Adami, Pasquale Del Gaudio

**Affiliations:** 1 Department of Pharmacy, University of Salerno, 84084 Fisciano, SA, Italy; 2 Immunology Department, Victor Babes National Institute of Pathology, 050096 Bucharest, Romania; 3 Department of Pharmacology, Pharmacy and Pharmaceutical Technology, R+D Pharma Group (GI-1645), Faculty of Pharmacy and Health Research Institute of Santiago de Compostela (IDIS), Universidade de Santiago de Compostela, E-15782 Santiago de Compostela, Spain; 4 Department of Physics “E. R. Caianiello”, University of Salerno, 84084 Fisciano, SA, Italy; 5 NanoMates Center, University of Salerno, 84084 Fisciano, SA, Italy

**Keywords:** aerogel capsules, prilling, supercritical drying, ketoprofen lysine, wound dressing

## Abstract

The production of aerogels for different applications has been widely known, but the use of polysaccharide-based aerogels for pharmaceutical applications, specifically as drug carriers for wound healing, is being recently explored. The main focus of this work is the production and characterization of drug-loaded aerogel capsules through prilling in tandem with supercritical extraction. In particular, drug-loaded particles were produced by a recently developed inverse gelation method through prilling in a coaxial configuration. Particles were loaded with ketoprofen lysinate, which was used as a model drug. The core-shell particles manufactured by prilling were subjected to a supercritical drying process with CO_2_ that led to capsules formed by a wide hollow cavity and a tunable thin aerogel layer (40 μm) made of alginate, which presented good textural properties in terms of porosity (89.9% and 95.3%) and a surface area up to 417.0 m^2^/g. Such properties allowed the hollow aerogel particles to absorb a high amount of wound fluid moving very quickly (less than 30 s) into a conformable hydrogel in the wound cavity, prolonging drug release (till 72 h) due to the in situ formed hydrogel that acted as a barrier to drug diffusion.

## 1. Introduction

The rising prevalence of non-communicable diseases (chronic illnesses) and the advancing age of the population have underscored the need for proper wound management and highlighted the substantial societal burden associated with wounds [1]. The most diffused wounds, such as diabetic foot ulcers, venous leg ulcers, and pressure sores, promote a chronic condition in the patient with long hospitalization, frequent changes of expensive dressings [2], and, consequently, a financial burden for them and/or health systems. One of the main drawbacks related to the healing of chronic wounds is the lack of an “ideal dressing” that is able to satisfy all the requirements necessary to promote the healing process [3,4] and, at the same time, it acts as a low-cost solution. A desirable wound dressing should demonstrate biocompatibility and biodegradability while also having the capacity to adapt to diverse wound shapes and depths, thus ensuring pain-free removal for patients. 

Furthermore, it should promote effective wound ventilation by facilitating the diffusion of oxygen and water vapor as well as proficiently manage excessive fluid in the case of exudating wounds [5]. Excessive exudate, while essential for maintaining a favorable wound environment and stimulating the healing process, can impede healing and serves as a conducive substrate for bacterial growth. Therefore, it is crucial to effectively manage and control exudate levels to optimize the wound healing process and minimize the risk of bacterial proliferation [6,7]. Wound dressings play a crucial role in creating a physical barrier that protects the wound environment from external contaminants. Additionally, they can incorporate active pharmaceutical ingredients (APIs) that facilitate a faster wound-healing process. However, in the case of advanced dressings, it is essential for them to carefully regulate the release of the loaded substances at the site of the wound. This controlled release mechanism ensures that the APIs are released in a controlled manner, providing targeted therapeutic effects while preventing any undesired consequences [8]. Nowadays, different commercially available dressings satisfy most of these properties, but they still present some adverse effects, such as local irritation or trauma after removal in the case of foam dressings; dehydration in the case of films; and occlusiveness in the case of hydrocolloids and frequent dressing changes typical of hydrogels [9,10]. The search for an optimal benchmark in wound dressings is an ongoing endeavor, resulting in the continual exploration of new materials. Among these, scientists are particularly interested in exploring polysaccharide-based polymers derived from natural sources, such as plants, animals, algae, and microorganisms. These biocompatible, biodegradable, non-toxic, and cost-effective excipients hold great potential for advancing wound dressing technology [11]. In situ gelling formulations incorporating aerogels offer a potential alternative to traditional dressings. These aerogels have recently been proposed for wound treatment due to their unique property of gelling exclusively at the site of the wound upon contact with wound fluids while remaining non-gelled on the surrounding healthy skin. This characteristic allows for a less traumatic removal process. Moreover, aerogels can enhance drug retention at the wound site and provide controlled release, facilitating easy, consistent, and precise administration of the required dosage [12,13]. Aerogels represent nanostructured substances featuring an immensely high level of porosity within the mesoporous range. These materials exhibit interconnected pores throughout their structure. They are formed by eliminating the liquid component from a gel and replacing it with a gas. This process effectively prevents pore collapse and preserves the integrity of the porous structure without compromising the wet material’s texture [14]. Multiple methods can be employed to obtain aerogels but the optimal approach is through supercritical drying. This technique utilizes supercritical fluids, with carbon dioxide (CO_2_) being the most commonly employed supercritical fluid due to its remarkable properties. Supercritical fluid extraction (SAE) is widely recognized as the preferred method for producing aerogels [15]. Additionally, aerogels showcase a multitude of exceptional characteristics, including notable pliability, transparency, reduced thermal conductivity, superior mechanical resilience, and an extremely low dielectric constant. These extraordinary properties render aerogels highly adaptable for a wide range of applications. They serve as efficient thermal insulators, find utility in aerospace manufacturing, act as catalysts, provide effective filtration and environmental remediation solutions, enable chemical sensing capabilities, contribute to Cherenkov counters, drive piezoelectric devices, facilitate acoustic transduction, empower microwave electronics, serve as molds for metal casting, offer water-repellent coatings, and support advancements in battery [16] and food-related technologies, packaging [17], and, in recent times, they have found utility in the field of medical and pharmaceutical applications, particularly in the realm of controlled delivery systems for nonsteroidal anti-inflammatory drugs [18,19], growth factors [20], steroidal anti-inflammatory drugs [21] from scaffolds, and chitosan in chronic wounds [22], among others. The scientific literature has already documented the advancement of utilizing polysaccharide-based aerogels as promising carriers for drug delivery systems. Significantly, monolithic structures have been successfully fabricated from a variety of raw materials to create aerogels. Examples include potato and corn-based starch aerogels [23]; pectin aerogels derived from citrus peels and available as both powders and monoliths [24]; calcium alginate aerogels formed into beads, microspheres, and monolithic forms [25,26]; as well as aerogel beads made from K-carrageenan [27] and agar [28]. Furthermore, monoliths have been produced using chitin [29,30] and chitosan [31,32] while chitosan beads [33] and cellulose monoliths from diverse sources [34,35] have also been investigated. Moreover, prior research has enabled the development of a novel inverse gelation technique conducted directly at the coaxial nozzle of the prilling apparatus. This method has been employed for the manufacturing of core-shell microparticles based on alginate, which serves as a carrier for various active pharmaceutical ingredients (APIs) with diverse properties [36]. When incorporated into gel capsules, these formulations present themselves as promising contenders to produce aerogel capsules as novel vehicles for drug delivery in the topical treatment of wounds. However, the feasibility of fabricating polysaccharide-based aerogels in the form of bead structures, featuring a hollow core to accommodate the active pharmaceutical ingredient (API) and enhance exudate adsorption capacity, coupled with a thin polymeric shell to optimize drug encapsulation, remains an unresolved matter to date. To overcome these challenges, the primary objective of this study was to develop loaded aerogel capsules using a combination of prilling and supercritical CO_2_ (sc-CO_2_) drying techniques. The production of core-shell microparticles is achieved through an inverse gelation methodology, wherein an O/W/O emulsion acts as the hydrophobic internal phase, while an alginate solution serves as the hydrophilic external phase. In this approach, hydrophobic core/hydrophilic shell microparticles are prepared and subsequently loaded with a non-steroidal anti-inflammatory drug (ketoprofen lysinate). Previous research has indicated that the short-term administration of these drugs can expedite the healing process of combat-related extremity wounds by modulating the levels of inflammatory cytokines [37].

## 2. Results and Discussion

Preliminary experiments were conducted to set operating conditions to produce core-shell particles with gelation taking place right out of the coaxial nozzle of the prilling apparatus.

In order to identify the most suitable value for achieving inverse gelation at the nozzle, a range of concentrations of aqueous CaCl_2_ solutions were employed during the preparation of the O/W/O emulsion. After thorough experimentation, it was discovered that a concentration of 13.4 g/L emerged as the optimal choice, demonstrating the ability to induce inverse gelation effectively, thus, enabling the formation of core-shell particles by gelling the alginate solution coming out of the outer channel of the coaxial nozzle. Different amounts of PVA and Span^®^ 85 were used as surfactants to prepare the emulsion. Overall, this process provided the optimizing values at 1.0% (*w*/*v*) for PVA and at 0.5% (*w*/*v*) for Span^®^ 85. In fact, the double emulsion prepared with such concentrations was sufficiently stable to be processed as well as sensible enough to the vibration-induced discharge at the exit of the nozzle to enable the interaction between calcium cations and the alginate solution coming out from the outer nozzle.

Different frequencies of vibration were also tested to identify values that are able to break up the jet coming out from the coaxial nozzle in uniform droplets.

Extensive experimentation was conducted to achieve the desired result of a spherical core enveloped by a homogeneous layer of alginate. Multiple factors were thoroughly examined, including the concentrations of alginate and ketoprofen lysinate, frequencies, nozzle diameters, the vertical distance between the nozzle and the gelling bath, and electrode voltage. Through meticulous testing, the most optimal combination of these parameters was determined [38]. Table 1 shows only some of the tested parameters, while all the parameters are reported in Table S1, as Supplementary Materials. The sphericity of the obtained particles as well as the layer thickness for each batch were evaluated based on a given score (zero or one). Spherical and homogeneous particles with a single core encapsulated by a homogeneous alginate shell were given the higher score, nominally one.

According to the findings presented in Table 1, the batches produced using coaxial nozzles with diameters of 300/700 μm and 450/600 μm, regardless of the use of the electrode, did not yield satisfactory particle results. Conversely, favorable outcomes were observed when utilizing a coaxial nozzle with a diameter of 450 μm for the inner layer and 900 μm for the outer layer. This particular configuration resulted in the formation of core-shell particles that exhibited desirable coaxiality, sphericity, and sizes exceeding 1 mm.

Notably, the use of the electrode did not have any significant impact on the production of particles of such sizes; therefore, it was not employed for further studies. As indicated in Table 1, the process of gelation in ethanolic media demonstrated superior encapsulation compared to gelation in aqueous media.

Moreover, it was observed that the reduction of the distance between the nozzle and the gelation bath from 10 to 3 cm led to particles being more homogeneous in terms of sphericity and coaxiality, probably due to the reduction of the falling time that promoted the lengthening of the drops [36]. In addition, a higher emulsion flow rate led to core-shell particles with a more homogeneous core, as in the case of higher alginate concentrations (1.75% and 2.25%, *w*/*v*).

**Figure 1:**
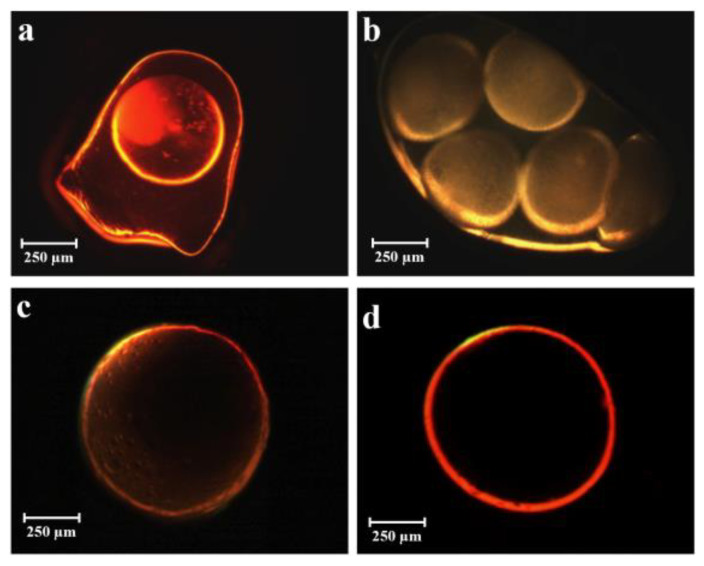
FM images of core-shell microparticles obtained with different conditions: (**a**) non-spherical and non-homogeneous, alginate coated oily particle (Table 1: # 19,20); (**b**) multi-core capsule obtained by non-optimized parameters (Table 1: # 11, 12, 13); (**c**,**d**) homogeneous capsule in terms of sphericity and coaxiality (Table 1: # from 26 to 51).

Figure 1 illustrates the confirmation of emulsion encapsulation within a thin alginate layer through fluorescent microscopy assays. Under optimized conditions, this led to the formation of homogeneous and spherical core-shell particles with well-defined single-centered cores. These particles were subsequently transformed into aerogels through supercritical carbon dioxide (sc-CO_2_) drying. After fine-tuning all the operational parameters, the addition of ketoprofen lysinate, a model drug, to the aqueous phase of the O/W/O emulsion resulted in the successful production of core-shell particles with the encapsulated drug. These particles were further subjected to drying using sc-CO_2_ to create loaded aerogel capsules. To ensure the elimination of any potential traces of water before the sc-CO_2_ drying process, a solvent exchange was performed twice. This step was crucial to maintain the integrity and quality of the final loaded aerogel capsules.

A preliminary set of parameters was established for sc-CO_2_ drying, utilizing a temperature of 40 °C, a pressure of 120 bar, and a processing time of 210 min. The resulting aerogels were subsequently analyzed using a nitrogen adsorption porosimeter to assess their textural properties. The BET-specific surface area (S_a_) measurements revealed a relatively low surface area ranging from 8.1 to 27.5 m^2^/g. Additionally, the BJH cumulative desorption pore volume (Vp_BJH,_ d) values fell within the range from 0.08 to 0.27 cm^3^/g. These measurements provided insights into the porosity and textural characteristics of the aerogels produced through sc-CO_2_ drying under the specified parameters.

**Figure 2:**
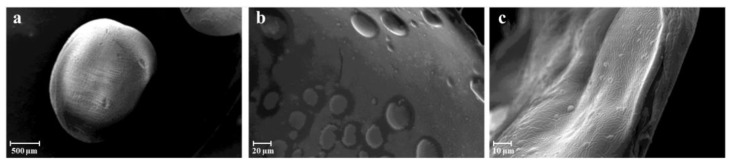
SEM pictures of aerogels following SC-CO_2_ drying (1) exhibit the following characteristics: (**a**) an individual bead, (**b**) the internal section of a bead, and (**c**) ketoprofen lysinate crystals within the inner region.

SEM (Scanning electron microscopy) images provided valuable insights into the effects of supercritical drying (1) on the capsules. It was observed that the sphericity of the capsules remained unaltered, as depicted in Figure 2a. However, the analysis of cryo-fractured capsules revealed the presence of craters in the internal alginate layer (Figure 2b). These craters were likely formed due to the removal of residual oil droplets from the emulsion during the prilling process. Furthermore, as depicted in Figure 2c, the SEM images revealed that ketoprofen crystals were skillfully incorporated into the polymeric matrix. This observation signifies that the solvent elimination achieved through supercritical drying effectively removed the oily phase without eliminating the loaded ketoprofen lysinate, although its concentration was significantly reduced.

Referring to the observed structural imperfections, as depicted in Figure 2, and textural properties obtained by nitrogen adsorption porosimeter, as well as a reduced load of the drug, a second set of operating parameters for sc-CO_2_ drying, namely sc-CO_2_ drying (2), was carried out. The operating temperature was kept at 40 °C while increasing the pressure up to 140 bar and the time of the drying process up to 270 min. The aerogels obtained with the second set of parameters displayed notable enhancements in specific surface area, pore volume, and mesoporous pore diameters, as demonstrated in Table 2. All formulations showed porosity ranging from 89.9% to 95.3%, which was higher in the case of the blank formulations and formulations loaded with 20% (*w*/*w*) of the drug, probably due to stronger removal of ketoprofen incorporated into the polymer matrix during the drying process [39]. For the same reason, density was higher in the case of aerogels made from 1.75% (*w*/*v*) alginate containing 5% (*w*/*w*) and 10% (*w*/*w*) ketoprofen lysinate and, as a consequence, their porosity was slightly lower. Moreover, the presence of ketoprofen lysinate in the aerogel formulations, obtained by the sc-CO_2_ drying (2), showed that drug content was higher compared to the homologous formulations obtained by sc-CO_2_ drying (1), with encapsulation efficiency ranging from 56% to 68% in the case of F1.75 and 74% in the case of F20, as reported in Table 2. 

**Figure 3:**
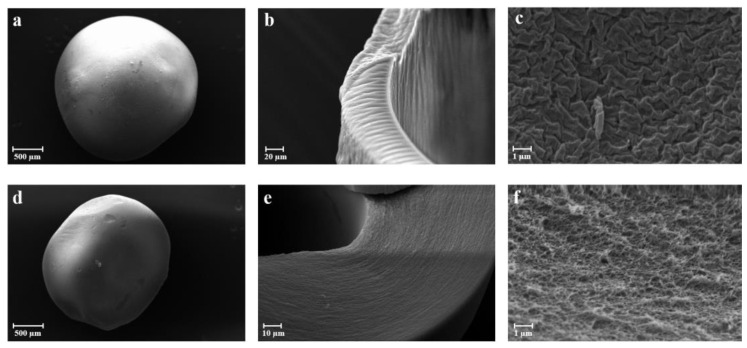
SEM images of F_1.75-10_2 (**a**–**c**) and F_1.75-20_2 (**d**–**f**) aerogels obtained after sc-CO_2_ drying (2) at different magnifications: (**a**,**d**) single bead; (**b**,**e**) shell layer; (**c**,**f**) porous structure of the matrix.

**Figure 4:**
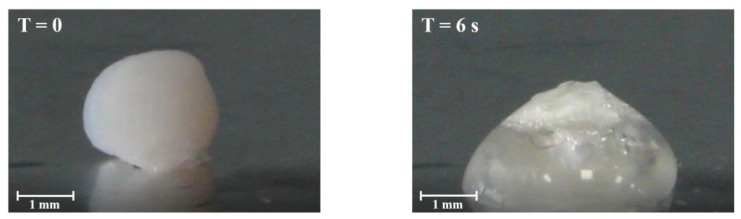
The fluid uptake behavior of the F_1.75-20_ 2 formulation undergoes a transition from aerogel to hydrogel when it comes into contact with a single droplet of simulated wound fluid. This transition is visually depicted through photographs taken at two time intervals: 0 s and 6 s.

The SEM images related to the sc-CO_2_ drying process (2) validate that the spherical shape of the aerogel particles remained unaffected by the supercritical drying conditions, as depicted in Figure 3a,d. Moreover, in this case, the capsules were cryo-fractured, highlighting a greater thickness of the shell layer (Figure 3c,f), probably due to the increase in the pressure of sc-CO_2_, and a greater porosity of the internal polymer matrix (Figure 4b,e), probably due to the removal of ketoprofen crystals embedded in the matrix, which in this case, is stronger than the sc-CO_2_ (1) process (Figure 3c) [23]. In particular, the differences observed between the capsules obtained with the sc-CO_2_ drying (1) and the sc-CO_2_ drying (2) processes might be related to the different densities of sc-CO_2_ induced by the increase of pressure. The change of density, in fact, affected both the solvent power and the surface tension of the sc-CO_2_. Specifically, the rise in pressure while maintaining a constant temperature resulted in an augmented density and reduced surface tension. This facilitated the creation of a polymer matrix with enhanced porosity that still retained ketoprofen.

The fluid uptake capacity of the aerogel capsules, produced through sc-CO_2_ drying (2), was assessed by measuring the weight ratio of the formed hydrogel to the aerogel at various time intervals after exposure to SWF [40]. Consistent with expectations, the fluid uptake was found to be directly proportional to the surface area and porosity of the aerogels [41]. The formulations displayed substantial weight increases, with F_1.75-10_2 experiencing a remarkable rise of approximately 480% and F_1.75-20_2 demonstrating an even higher increase of around 505% compared to their initial weights. The greater absorption of liquid by F_1.75-20_2, related to the hydrogel weight increase, confirmed that a higher concentration of the drug model leads to a greater porosity, as visible in Table 2, where the porosity of this batch is 93.1%, with a pore volume of 2.35 ± 0.12 cm^3^/g compared to 89.9% of F_1.75-10_2 that had a pore volume of 1.96 ± 0.10 cm^3^/g. Moreover, F_1.75-10_2 and F_1.75-20_2 showed, respectively, surface areas of 242.7 ± 12.1 m^2^/g and 368.4 ± 18.4 m^2^/g, representing values that are much greater than the surface areas of the capsules obtained with sc-CO_2_ drying (1), where the surface area and porosity did not exceed the values for the sc-CO_2_ drying (2) process had respective, values of 27.5 m^2^/g and 0.30 cm^3^/g. These values indicate a poor absorption capacity of the liquid from part of the capsules obtained by sc-CO_2_ drying (1). In addition, in Figure 4, it is possible to observe the shrinking of the particles before the gelling phase. The occurrence of this phenomenon can be attributed to the partial degradation of the alginate shell, which becomes more pronounced and occurs at a faster rate in formulations with larger surface areas and greater porosity [42]. Even though the gelling rate was very fast for all formulations (less than 30 s), the total gelling time was reduced to 12 s for the F_1.75-20_2 aerogel capsules.

**Figure 5:**
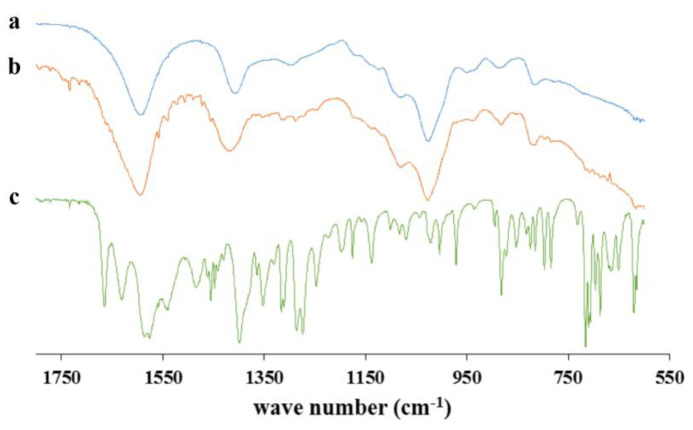
FTIR-ATR spectra of the alginate, the pure ketoprofen, and the loaded-aerogel; (a) alginate, (b) F_1.75-20_2 aerogel formulation, and (c) ketoprofen lysinate.

The results obtained by infrared spectroscopy (FTIR-ATR) [43] are shown in Figure 5, reporting the spectra of the pure ketoprofen, the alginate, and the loaded aerogel containing 20% of ketoprofen. Two peaks were observed in the stretching region (C=O) for the pure ketoprofen, between 1667 and 1555 cm^−1^, which represents the vibrational stretching of the carboxylic group and the vibrational stretching of the carbonyl in the ketonic group, respectively [44]. In the loaded aerogels, these peaks were not presented, and this could be due to its shift to lower wavelength numbers being covered by the stretching of the alginate carboxylic group, caused by the formation of hydrogen bonds between the alginate and the ketoprofen and the formation of interactions between the hydroxyl groups of ketoprofen and the Ca^2+^ present in the gel matrix of the polymeric particles [45].

**Figure 6:**
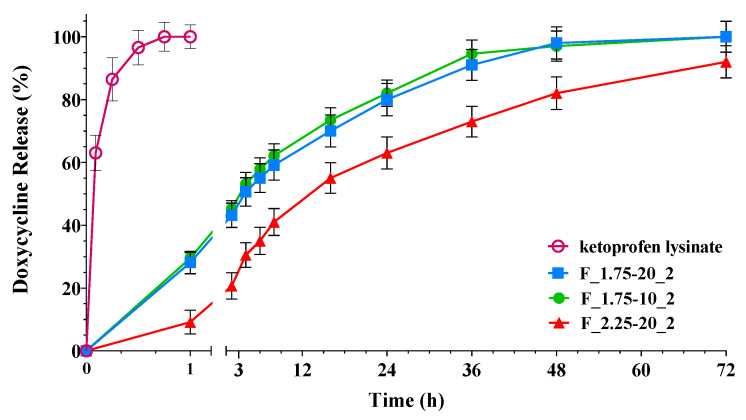
Release profiles of ketoprofen-loaded aerogel capsules with different polymer amounts and ketoprofen load: All samples, except the ketoprofen lysinate indicated by the empty circle, are the formulations (F) obtained with sc-CO_2_ drying (2). In particular, 10 and 20 are the % (*w*/*w*) concentrations of ketoprofen lysinate and 1.75 and 2.25 are the % (*w*/*v*) concentrations of alginate.

To evaluate the release characteristics of the encapsulated drug, in vitro release experiments were performed using Franz-type vertical diffusion cells. The acceptor fluid used in these experiments was simulated wound fluid (SWF). Figure 6 showcases illustrative release curves of ketoprofen from hollow aerogel particles generated with varying amounts of polymer.

Figure 6 demonstrates that pure ketoprofen lysinate achieved complete release in less than 45 min, whereas the formulations extended the release over 72 h. Aerogel particles exhibited an initial burst effect, releasing 40–60% of the drug within the first 9 h. The release rate decreased with higher amounts of alginate in the formulations. The prolonged release in high-alginate formulations can be attributed to the ketoprofen–alginate interactions observed in the FT-IR studies (Figure 5) and the increased consistency of in situ hydrogel formation.

## 3. Conclusions

This work reports the development of a robust method for producing biomolecule- loaded core-shell beads using the prilling technique, combined with supercritical drying, for future applications in wound healing. The resulting aerogels exhibit remarkable properties, including a narrow size distribution, high surface area (up to 417.0 ± 20.8 m^2^/g), and good porosity (up to 95.3%), making them good candidates for drug encapsulation and controlled release. The loading of ketoprofen lysinate, used as a model drug, showed excellent encapsulation efficiency for sc-CO_2_ dried formulations (up to 74%). Furthermore, the aerogel formulations demonstrate a very interesting ability to absorb a significant amount of exudate and being able to transform into a soft hydrogel, effectively prolonging drug release for up to 72 h. These findings suggest that alginate aerogel capsules have the potential to serve as a self-consistent dosage form, capable of both controlling drug release and efficiently managing wound exudate, particularly in the treatment of acute and chronic wounds. The novel approach presented in this work paves the way for further research and the development of advanced therapeutic systems with improved patient outcomes and enhanced medical applications. The alginate aerogel capsules can be explored for personalized medicine, where tailored doses and drug releases can be designed to meet the unique requirements of individual patients.

## 4. Materials and Methods

### 4.1. Materials

Medium viscosity sodium alginate (MMW, 120 kDa, 1% viscosity, 40 mPa·s, mannuronic acid content of 70%) and ketoprofen lysinate were kindly donated by Dompè Pharma (Dompè S.p.A., L’Aquila, Italy). The sunflower seed oil was purchased from Oleificio del Golfo (Latina, Italy). Furthermore, Span^®^ 85 and polyvinyl alcohol (PVA) were purchased from Sigma-Aldrich (Milan, Italy). Calcium chloride, CaCl_2_ (93% purity), was also obtained from Sigma-Aldrich (Milan, Italy), while Dichloromethane was purchased from Carlo Erba (Milan, Italy). Absolute ethanol was purchased from VWR (Llinars del Vallés, Spain). Finally, Carbon dioxide (99.8% purity) was supplied from Praxair (Madrid, Spain). All other reagents were purchased from Sigma-Aldrich (Milan, Italy) and employed as received.

### 4.2. Methods

#### 4.2.1. Production of Core-Shell Microparticles

For the manufacturing of core-shell loaded-microparticles, aqueous alginate solutions at different concentrations (1.50–2.25% (*w*/*v*)) were used to form the shell of the gel beads and an O/W/O emulsion was used to form the core. The alginate capsules were prepared using a prilling apparatus (Encapsulator B-360, Büchi Labortechnik AG, St. Gallen, Switzerland) to test different values of inner/outer nozzle diameter, flow rates, vibration frequencies, electrode, and amplitude. Different amounts of ketoprofen lysinate were dissolved into the aqueous phase of the O/W/O emulsion in order to obtain 5%, 10%, and 20% (*w*/*w*) drug-loaded emulsions. The obtained gel beads were collected in a 0.3 M CaCl_2_ aqueous or ethanolic solution placed at different distances (3–5 cm) from the nozzle exit and maintained under gentle stirring for 5 min before being rinsed and collected in water or absolute ethanol, respectively. (More specific information about operating conditions are reported as Supplementary Materials).

#### 4.2.2. Supercritical Drying of Core-Shell Particles

Before the sc-CO_2_ drying process, a solvent exchange in the capsules was carried out twice in order to eliminate the possible traces of water. The sc-CO_2_ drying was carried out in a 100 mL stainless steel autoclave (Thar Technologies, Pittsburg, PA, USA) at 40 °C and a CO_2_ flow rate of 7 g/min during the first hour and at 5 g/min during the rest of the process. Different assays were carried out at 120 bar during 210 min (sc-CO_2_ drying 1), and at 140 bar during 270 min (sc-CO_2_ drying 2) into the process for the production of void aerogels loaded with ketoprofen lysinate.

#### 4.2.3. Characterization of the Aerogels

##### Fluorescent Microscopy

To analyze the shape and structure of participles, we conducted fluorescent microscopy (FM) assays by analyzing core-shell particles in a ZeissAxiophot fluorescent microscope with an Apochroma 20 × 1.4 NA no oil immersion objective (Carl ZeissVision, München-Hallbergmoos, Germany) using standard optic filters.

##### Scanning Electron Microscopy

The morphology and size distribution of the aerogel capsules was studied by scanning electron microscopy (SEM) using a Carl Zeiss EVO MA10 microscope provided with a secondary electron detector (Carl Zeiss SMT Ltd., Cambridge, UK) and equipped with a LEICA EMSCD005 metallizator producing a deposition of 200–400 Å thick gold layer. The aerogel samples were dispersed on a carbon tab previously stuck to an aluminum stub (Agar Scientific, Stansted, UK). The samples were then coated with a gold layer using a sputter coater (mod. 108 Å, Agar Scientific, Stansted, UK) at 35 mA for 155 s. At least 20 SEM images were taken into account for each sample.

##### Nitrogen Adsorption Porosimetry

The textural properties of aerogels were studied by nitrogen adsorption–desorption analysis in an ASAP 2000 apparatus (Micromeritics, Norcross, GA, USA). The specific surface area (S_a_) and pore size distribution of aerogel and cryo-gel particles were calculated by the Brunauer–Emmett–Teller (BET) and the Barret, Joyner, and Halenda (BJH) methods, respectively. The overall specific pore volume (Vp_BJH,d_) and the mean pore diameter (Dp_BJH,d_) were also obtained by the BJH method. Samples were previously outgassed (<10^−5^ mbar) at 60 °C for at least 20 h before the analysis.

##### FTIR-ATR Spectroscopy

Infrared analyses were performed in an FT-IR/NIR Frontier Spectrophotometer (Perker Elmer, MA, USA) equipped with a single reflection horizontal ATR accessory having a diamond coated ZnSe top-plate crystal fixed at an incident angle of 90° (Universal ATR Accessory, Perkin Elmer, MA, USA). The samples were analyzed using 64 scans with a 1 cm^−1^ resolution step.

##### Drug Content and Encapsulation Efficiency of Aerogels

The drug content and the encapsulation efficiency analysis of ketoprofen lysinate-loaded aerogels were carried out by the UV–Vis spectrophotometer (Evolution 201 UV-Vis Double Beam Spectrophotometer Thermo Scientific, Waltham, MA, USA) at a wavelength of 254 nm. Approximately 10 mg of each formulation were added to 2.5 mL PBS (100 mM, pH 7.4) under vigorous stirring conditions for 30 min and then filtered and centrifuged at 6000 rpm for 30 min. After that, the supernatant was measured with the spectrophotometer. Each analysis was performed in triplicate and the drug content, and the encapsulation efficiency were calculated by Equations (1) and (2), respectively.

(1) $$Drug\;content\;\%=\frac{experimental\;amount\;of\;drug}{weighted\;amount\;of\;powder}\times 100$$

(2) $$Encapsulation\;efficiency\;\%=\frac{experimental\;amount\;of\;drug}{theoretical\;amount\;of\;drug}\times 100$$

##### Volume and Density Measurements

Aerogel capsules density was calculated by the following Equation (3):

(3) $$\frac{1}{\rho }=\frac{Vtotal}{g}=\frac{1}{\rho skeletal}+\frac{1}{\rho envelope}+\frac{Vmacropore}{g}$$

where 1/ρenvelope is the desorption pore volume (cm^3^/g) obtained by the BJH method. The shell thickness and the particle radius were obtained using image analyses and allowed the calculation of different volumes (macropore, mesopore, and solid volumes). The skeletal density (ρskeletal) of aerogels was measured by a helium pycnometer (Micropycnometer Quanta-Chrome MPY-2, Boynton Beach, FL, USA) at room temperature using helium as the displacement gas. A sealed chamber of the known volume containing a previously weighted amount of sample was pressurized with helium to 1.20 bar and, once stabilized, the pressure was recorded. Then, a valve was opened to expand the gas into a reference chamber of known volume and this pressure was also subsequently recorded when stabilized. This pressure drop ratio was compared to the behavior of the system when a known volume standard underwent the same process. The helium pycnometer was previously calibrated using two metallic spheres of known weight and volume.

In consequence, the overall porosity was calculated from Equation (4):

(4) $$\varepsilon \left(\%\right)=\left(\frac{\frac{Vmacropore}{g}+\frac{1}{\rho envelope}}{\frac{1}{\rho }}\right)\times 100$$

##### Fluid Uptake

The fluid uptake ability of the aerogel capsules was evaluated as the weight ratio of the formed hydrogel and starting aerogel and at different time points when formulations were in contact with simulated wound fluid (SWF) composed of a 50% maximum recovery diluent (MRD) (Sigma Aldrich, Milan, Italy), containing 0.1% (w/v) peptone (peptide digestion of animal tissue), 0.9% (w/v) chloride of sodium, and a 50% fetal calf serum (Sigma Aldrich, Milan, Italy) [46]. The weight of the gel formed was measured at regular intervals until it reached constant weight. All experiments were performed in triplicate.

##### Drug Release Studies

Drug release experiments were conducted in vertical Franz-type diffusion cells (Disa, Milan, Italy), with an exposed surface area of 0.6 cm^2^. A cellulose acetate filter with a pore size of 0.45µm (Sartorius, Goettingen, Germany) was used as support for the in situ formed hydrogel obtained by the transformation of the aerogel particles. The donor compartment was filled with approximately 30 mg of ketoprofen lysinate-loaded aerogels and 200 µL of the receptor phase to promote the formation of the gel. The receptor phase was SWF, thermostated at 37 °C, and magnetically stirred to prevent any boundary layer effects. The receptor solution was sampled and analyzed by UV spectrophotometer at λ = 259 nm (Lambda 25 UV/VIS Spectrometer, PerkinElmer, Waltham, MA, USA) for the determination of ketoprofen released at the donor compartment. Tests were conducted on six different batches of particles and mean values, and standard deviations were reported.

## Figures and Tables

**Table 1:** Prilling process parameters. F: Frequency; FR_ALG_ and FR_emulsion_: flow rates of the alginate solution and the emulsion, respectively; H_2_O and EtOH: aqueous and ethanolic gelling baths, respectively. For the batches indicated in this table, the values of frequency and amplitude are not given because they are identical for all batches (340 Hz and 5, respectively).

Batch (#)	ALG % (*w*/*v*)	Ketoprofen Lys % (*w*/*w*)	FR_ALG_ (Bar)	FR_emulsion_ (mL/min)	Inner/Outer Nozzle (μm)	Falling Distance (cm)	Gelling Bath (CaCl_2_ 0.3 M)	Sphericity	Coaxiality
11	1.5	-	100	5.00	450/600	10	H_2_O	0	0
12		-	84					0	0
13		-	54					1	0
19	1.75	-	140	4.87	300/700	4.5	H_2_O	0	0
20		-	96	5.00				0	0
26	1.75	-	96	6.00	450/900	4.5	EtOH	1	0
34		-				3.0		1	1
48	2.25	-	150					1	1
45	1.75	5.0	96	6.00	450/900	3.0	EtOH	1	1
39		10.0						1	1
42		20.0						1	1
49	2.25	5.0	150					1	1
50		10.0						1	1
51		20.0						1	1

**Table 2:** Textural properties of formulations after sc-CO_2_ drying (1) and sc-CO_2_ drying (2) protocols.

Sample	Alg % (*w*/*v*)	Ketoprofen Lys. % (*w*/*w*)	Surface Area (S_a_) (m^2^/g)	Pore Volume (Vp_BJH, d_) (cm^3^/g)	Pore Diameter (Dp_BJH, d_) (nm)	Density (g/cm^3^)	Porosity (%)	E.E. (%)
F_1.75-0_1	1.75	-	19.3 ± 2.1	0.27 ± 0.05	11.16 ± 1.22	0.357	82.4	-
F_1.75-5_1		5	27.5 ± 3.2	0.21 ± 0.03	10.10 ± 1.16	0.313	85.8	9.2
F_1.75-10_1		10	8.1 ± 1.2	0.18 ± 0.03	10.36 ± 1.43	0.391	80.4	10.1
F_1.75-20_1		20	26.8 ± 6.6	0.11 ± 0.04	11.75 ± 0.99	0.298	84.2	14.4
F_2.25-0_1	2.25	-	21.2 ± 4.2	0.20 ± 0.05	10.51 ± 1.28	0.327	83.6	-
F_2.25-20_1		20	20.1 ± 3.2	0.08 ± 0.03	12.35 ± 1.58	0.384	83.2	32.3
F_1.75-0_2	1.75	-	344.9 ± 17.2	1.40 ± 0.07	16.83 ± 0.84	0.073	95.3	-
F_1.75-5_2		5	369.6 ± 18.5	1.45 ± 0.07	14.24 ± 0.71	0.146	91.1	56.3
F_1.75-10_2		10	242.7 ± 12.1	1.96 ± 0.10	25.37 ± 1.27	0.107	89.9	61.2
F_1.75-20_2		20	368.4 ± 18.4	2.35 ± 0.12	25.62 ± 1.28	0.068	93.1	68.5
F_2.25-0_2	2.25	-	238.2 ± 11.9	1.46 ± 0.07	20.51 ± 1.03	0.072	94.1	-
F_2.25-20_2		20	417.0 ± 20.8	2.97 ± 0.15	27.14 ± 1.36	0.069	93.5	74.2

## Data Availability

Not applicable.

## Supplementary Materials

The following supporting information can be downloaded at https://www.mdpi.com/article/10.3390/gels9060492/s1. Table S1: Parameters of prilling process parameters tested for the production of core-shell particles. F is the Frequency, FRALG and FRemulsion are respectively the flow rates of the alginate solution and the emulsion, A is amplitude, and H_2_O and EtOH are gelling baths (respectively aqueous and ethanolic).

## References

[1] Bernardes B.G., Del Gaudio P., Alves P., Costa R., García-Gonzaléz C.A., Oliveira A.L. (2021). Bioaerogels: Promising nanostructured materials in fluid management, healing and regeneration of wounds. Molecules.

[2] Järbrink K., Ni G., Sönnergren H., Schmidtchen A., Pang C., Bajpai R., Car J. (2017). The humanistic and economic burden of chronic wounds: A protocol for a systematic review. Syst. Rev..

[3] Rezvani Ghomi E., Khalili S., Nouri Khorasani S., Esmaeely Neisiany R., Ramakrishna S. (2019). Wound dressings: Current advances and future directions. J. Appl. Polym. Sci..

[4] Broussard K.C., Powers J.G. (2013). Wound dressings: Selecting the most appropriate type. Am. J. Clin. Dermatol..

[5] Amante C., Esposito T., Del Gaudio P., Di Sarno V., Porta A., Tosco A., Russo P., Nicolais L., Aquino R.P. (2021). A novel three-polysaccharide blend in situ gelling powder for wound healing applications. Pharmaceutics.

[6] Guo S.A., DiPietro L.A. (2010). Factors affecting wound healing. J. Dent. Res..

[7] Bowler P., Duerden B., Armstrong D.G. (2001). Wound microbiology and associated approaches to wound management. Clin. Microbiol. Rev..

[8] Banjare J., Bhalerao S. (2016). Obesity associated noncommunicable disease burden. Int. J. Health Allied Sci..

[9] Han G., Ceilley R. (2017). Chronic wound healing: A review of current management and treatments. Adv. Ther..

[10] Boateng J.S., Matthews K.H., Stevens H.N., Eccleston G.M. (2008). Wound healing dressings and drug delivery systems: A review. J. Pharm. Sci..

[11] Yu Y., Shen M., Song Q., Xie J. (2018). Biological activities and pharmaceutical applications of polysaccharide from natural resources: A review. Carbohydr. Polym..

[12] De Cicco F., Porta A., Sansone F., Aquino R.P., Del Gaudio P. (2014). Nanospray technology for an in situ gelling nanoparticulate powder as a wound dressing. Int. J. Pharm..

[13] Van Tomme S.R., Storm G., Hennink W.E. (2008). In situ gelling hydrogels for pharmaceutical and biomedical applications. Int. J. Pharm..

[14] García-González C.A., Sosnik A., Kalmár J., De Marco I., Erkey C., Concheiro A., Alvarez-Lorenzo C. (2021). Aerogels in drug delivery: From design to application. J. Control. Release.

[15] Adami R., Russo P., Amante C., De Soricellis C., Della Porta G., Reverchon E., Del Gaudio P. (2022). Supercritical antisolvent technique for the production of breathable naringin powder. Pharmaceutics.

[16] Stergar J., Maver U. (2016). Review of aerogel-based materials in biomedical applications. J. Sol Gel Sci. Technol..

[17] Mikkonen K.S., Parikka K., Ghafar A., Tenkanen M. (2013). Prospects of polysaccharide aerogels as modern advanced food materials. Trends Food Sci. Technol..

[18] Franco P., De Marco I. (2020). Supercritical CO_2_ adsorption of non-steroidal anti-inflammatory drugs into biopolymer aerogels. J. CO2 Util..

[19] Lovskaya D., Lebedev A., Menshutina N. (2015). Aerogels as drug delivery systems: In vitro and in vivo evaluations. J. Supercrit. Fluids.

[20] Zhu Y., Zeng Q., Zhang Q., Li K., Shi X., Liang F., Han D. (2020). Temperature/near-infrared light-responsive conductive hydrogels for controlled drug release and real-time monitoring. Nanoscale.

[21] Goimil L., Jaeger P., Ardao I., Gómez-Amoza J.L., Concheiro A., Alvarez-Lorenzo C., García-González C.A. (2018). Preparation and stability of dexamethasone-loaded polymeric scaffolds for bone regeneration processed by compressed CO_2_ foaming. J. CO2 Util..

[22] Batista M., Gonçalves V.S., Gaspar F., Nogueira I., Matias A.A., Gurikov P. (2020). Novel alginate-chitosan aerogel fibres for potential wound healing applications. Int. J. Biol. Macromol..

[23] Mehling T., Smirnova I., Guenther U., Neubert R.H. (2009). Polysaccharide-based aerogels as drug carriers. J. Non Cryst. Solids.

[24] White R.J., Budarin V.L., Clark J.H. (2010). Pectin-derived porous materials. Chem. Eur. J..

[25] Chen K., Zhang H. (2019). Alginate/pectin aerogel microspheres for controlled release of proanthocyanidins. Int. J. Biol. Macromol..

[26] Ouyang X.-K., Zhao L., Jiang F., Ling J., Yang L.-Y., Wang N. (2022). Cellulose nanocrystal/calcium alginate-based porous microspheres for rapid hemostasis and wound healing. Carbohydr. Polym..

[27] Quignard F., Valentin R., Di Renzo F. (2008). Aerogel materials from marine polysaccharides. N. J. Chem..

[28] Robitzer M., Tourrette A., Horga R., Valentin R., Boissière M., Devoisselle J.-M., Di Renzo F., Quignard F. (2011). Nitrogen sorption as a tool for the characterisation of polysaccharide aerogels. Carbohydr. Polym..

[29] Robitzer M., Di Renzo F., Quignard F. (2011). Natural materials with high surface area. Physisorption methods for the characterization of the texture and surface of polysaccharide aerogels. Microporous Mesoporous Mater..

[30] Tsioptsias C., Michailof C., Stauropoulos G., Panayiotou C. (2009). Chitin and carbon aerogels from chitin alcogels. Carbohydr. Polym..

[31] Chang P.R., Jian R., Yu J., Ma X. (2010). Fabrication and characterisation of chitosan nanoparticles/plasticised-starch composites. Food Chem..

[32] Ko E., Kim H. (2020). Preparation of chitosan aerogel crosslinked in chemical and ionical ways by non-acid condition for wound dressing. Int. J. Biol. Macromol..

[33] El Kadib A., Molvinger K., Cacciaguerra T., Bousmina M., Brunel D. (2011). Chitosan templated synthesis of porous metal oxide microspheres with filamentary nanostructures. Microporous Mesoporous Mater..

[34] Liebner F., Haimer E., Wendland M., Neouze M.A., Schlufter K., Miethe P., Heinze T., Potthast A., Rosenau T. (2010). Aerogels from unaltered bacterial cellulose: Application of scCO_2_ drying for the preparation of shaped, ultra-lightweight cellulosic aerogels. Macromol. Biosci..

[35] Innerlohinger J., Weber H.K., Kraft G. (2006). Aerocellulose: Aerogels and aerogel-like materials made from cellulose. Macromol. Symp..

[36] Rodríguez-Dorado R., Landín M., Altai A., Russo P., Aquino R.P., Del Gaudio P. (2018). A novel method for the production of core-shell microparticles by inverse gelation optimized with artificial intelligent tools. Int. J. Pharm..

[37] Lisboa F.A., Bradley M.J., Hueman M.T., Schobel S.A., Gaucher B.J., Styrmisdottir E.L., Potter B.K., Forsberg J.A., Elster E.A. (2017). Nonsteroidal anti-inflammatory drugs may affect cytokine response and benefit healing of combat-related extremity wounds. Surgery.

[38] Posbeyikian A., Tubert E., Bacigalupe A., Escobar M.M., Santagapita P.R., Amodeo G., Perullini M. (2021). Evaluation of calcium alginate bead formation kinetics: An integrated analysis through light microscopy, rheology and microstructural SAXS. Carbohydr. Polym..

[39] Champeau M., Thomassin J.-M., Tassaing T., Jérôme C. (2015). Drug loading of polymer implants by supercritical CO_2_ assisted impregnation: A review. J. Control. Release.

[40] Nurzynska A., Klimek K., Palka K., Szajnecki Ł., Ginalska G. (2021). Curdlan-Based Hydrogels for Potential Application as Dressings for Promotion of Skin Wound Healing—Preliminary In Vitro Studies. Materials.

[41] Deuber F., Mousavi S., Federer L., Adlhart C. (2017). Amphiphilic nanofiber-based aerogels for selective liquid absorption from electrospun biopolymers. Adv. Mater. Interfaces.

[42] Malektaj H., Drozdov A.D., Declaville Christiansen J. (2023). Swelling of Homogeneous Alginate Gels with Multi-Stimuli Sensitivity. Int. J. Mol. Sci..

[43] Rodriguez Dorado R. (2019). Development of Technological Approaches Based on Supercritical Fluids for the Production of Polymeric Micro-nano Particulate Systems for Wound Healing. Ph.D. Thesis.

[44] Azeez A.M., Fakhre N.A. (2022). Determination of ketoprofen in tablet dosage forms by derivative IR spectroscopy. Egypt. J. Chem..

[45] Papageorgiou S.K., Kouvelos E.P., Favvas E.P., Sapalidis A.A., Romanos G.E., Katsaros F.K. (2010). Metal–carboxylate interactions in metal–alginate complexes studied with FTIR spectroscopy. Carbohydr. Res..

[46] Amante C., Andretto V., Rosso A., Augusti G., Marzocco S., Lollo G., Del Gaudio P. (2023). Alginate-pectin microparticles loaded with nanoemulsions as nanocomposites for wound healing. Drug Deliv. Transl. Res..

