# Supplementary material for: Hollow Particles Obtained by Prilling and Supercritical Drying as a Potential Conformable Dressing for Chronic Wounds
Source: Gels. 2023 Jun 16;9(6):492. doi: 10.3390/gels9060492 (PMC10298422; doi:10.3390/gels9060492)
Supplement: Supplementary file 1 [file gels-09-00492-s001.zip › gels-2436882-supplementary.pdf]

gels

MDPI

**Supplementary materials**

**Table S1**. All parameters prilling process parameters tested for the production of core-shell particles. F is the Frequency, $FR_{ALG}$ and $FR_{emulsion}$ are respectively the flow rates of the alginate solution and the emulsion, A is amplitude, and $H_2O$ and EtOH are gelling baths (respectively aqueous and ethanolic).

| Batch (#) | ALG (w/v) % | Keto-profen Lysinate (w/w) % | $FR_{ALG}$ (Bar) | $FR_{emulsion}$ (mL/min) | F (Hz) | Inner/outer nozzle (µm) | Falling distance (cm) | Electrode (V) | Gelling bath ($CaCl_2$ 0.3M) | A | Sphericity | Co-axiality |
|---|---|---|---|---|---|---|---|---|---|---|---|---|
| 1 | 1.5 | - | 47 | 4.87 | 300 | 450/900 | 15 | - | | | 0 | 0 |
| 2 | 1.5 | - | 90 | 4.87 | 340 | 450/900 | 15 | - | | | 0 | 0 |
| 3 | 1.5 | - | 75 | 4.87 | 340 | 450/900 | 15 | - | | | 1 | 0 |
| 4 | 1.5 | - | 60 | 4.87 | 340 | 450/900 | 15 | - | | | 0 | 0 |
| 5 | 1.5 | - | 88 | 4.87 | 320 | 450/900 | 15 | - | | | 0 | 0 |
| 6 | 1.5 | - | 54 | 4.87 | 280 | 450/900 | 15 | - | | | 0 | 0 |
| 7 | 1.5 | - | 100 | 4.87 | 340 | 450/900 | 15 | - | | | 0 | 0 |
| 8 | 1.5 | - | 75 | 4.87 | 340 | 450/900 | 10 | - | | | 0 | 0 |
| 9 | 1.5 | - | 60 | 4.87 | 340 | 450/900 | 10 | - | | | 0 | 0 |
| 10 | 1.5 | - | 100 | 4.87 | 340 | 450/900 | 10 | - | | | 0 | 0 |
| 11 | 1.5 | - | 100 | 5.00 | 340 | 450/600 | 10 | - | | | 0 | 0 |
| 12 | 1.5 | - | 84 | 5.00 | 340 | 450/600 | 10 | - | | | 0 | 0 |
| 13 | 1.5 | - | 54 | 5.00 | 340 | 450/600 | 10 | - | | | 1 | 0 |
| 14 | 1.5 | - | 75 | 4.87 | 340 | 450/900 | 4.5 | 500 | | | 0 | 0 |
| 15 | 2.0 | - | 75 | 4.87 | 340 | 450/900 | 4.5 | 1000-1500 | $H_2O$ | 5 | 0 | 0 |
| 16 | 2.0 | - | 120 | 4.87 | 340 | 450/900 | 4.5 | 1500-2000 | | | 0 | 0 |
| 17 | 1.75 | - | 101 | 4.87 | 340 | 450/900 | 4.5 | 2500 | | | 0 | 0 |
| 18 | 1.75 | - | 112 | 4.87 | 340 | 450/900 | 4.5 | 2000-2500 | | | 0 | 0 |
| 19 | 1.75 | - | 140 | 4.87 | 340 | 300/700 | 4.5 | 2500 | | | 0 | 0 |
| 20 | 1.75 | - | 96 | 5.00 | 340 | 300/700 | 4.5 | 2500 | | | 0 | 0 |
| 21 | 1.75 | - | 75 | 4.87 | 340 | 450/900 | 4.5 | - | | | 1 | 0 |
| 22 | 1.75 | - | 96 | 4.87 | 340 | 450/900 | 4.5 | - | | | 1 | 0 |
| 23 | 1.75 | - | 96 | 4.87 | 360 | 450/900 | 4.5 | - | | | 1 | 0 |
| 24 | 1.75 | - | 96 | 4.87 | 400 | 450/900 | 4.5 | - | | | 0 | 0 |
| 25 | 1.75 | - | 124 | 6.00 | 340 | 450/900 | 4.5 | - | | | 1 | 0 |
| 26 | 1.75 | - | 96 | 6.00 | 340 | 450/900 | 4.5 | - | | | 1 | 0 |
| 27 | 1.75 | - | 96 | 6.00 | 340 | 450/900 | 5.0 | - | | | 0 | 0 |
| 28 | 1.75 | - | 96 | 6.00 | 340 | 450/900 | 5.0 | 2500 | | | 0 | 0 |

| | | | | | | | | | | | | |
|---|---|---|---|---|---|---|---|---|---|---|---|---|
| 29 | 1.75 | - | 96 | 6.00 | 360 | 450/900 | 4.5 | - | | | 0 | 0 |
| 30 | 1.75 | 10.0 | 96 | 6.00 | 340 | 450/900 | 4.5 | - | | | 1 | 0 |
| 31 | 1.75 | 10.0 | 96 | 6.00 | 340 | 450/900 | 5.0 | - | | | 1 | 0 |
| 32 | 1.75 | 10.0 | 124 | 6.00 | 340 | 450/900 | 4.5 | 2500 | | | 0 | 0 |
| 33 | 1.75 | 1.0 | 96 | 6.00 | 340 | 450/900 | 4.5 | - | | | 1 | 0 |
| 34 | 1.75 | - | 96 | 6.00 | 340 | 450/900 | 3.0 | - | | | 1 | 1 |
| 35 | 1.75 | - | 75 | 4.87 | 340 | 450/900 | 3.0 | - | | | 0 | 0 |
| 36 | 1.75 | - | 75 | 6.00 | 340 | 450/900 | 3.0 | - | | | 0 | 0 |
| 37 | 1.75 | 5.0 | 96 | 6.00 | 340 | 450/900 | 4.5 | - | | | 0 | 0 |
| 38 | 1.75 | 5.0 | 75 | 6.00 | 340 | 450/900 | 3.0 | - | | | 0 | 0 |
| 39 | 1.75 | 10.0 | 96 | 6.00 | 340 | 450/900 | 3.0 | - | | | 1 | 1 |
| 40 | 1.75 | 10.0 | 75 | 4.87 | 340 | 450/900 | 3.0 | - | | | 0 | 0 |
| 41 | 1.75 | 20.0 | 96 | 6.00 | 340 | 450/900 | 4.5 | - | | 5 | 0 | 0 |
| 42 | 1.75 | 20.0 | 96 | 6.00 | 340 | 450/900 | 3.0 | - | EtOH | | 1 | 1 |
| 43 | 1.75 | 25.0 | 96 | 6.00 | 340 | 450/900 | 4.5 | - | | | 1 | 0 |
| 44 | 1.75 | 25.0 | 96 | 6.00 | 340 | 450/900 | 3.0 | - | | | 1 | 0 |
| 45 | 1.75 | 5.0 | 96 | 6.00 | 340 | 450/900 | 3.0 | - | | | 1 | 1 |
| 46 | 2.25 | - | 105 | 6.00 | 340 | 450/900 | 3.0 | - | | | 0 | 0 |
| 47 | 2.25 | - | 120 | 4.87 | 340 | 450/900 | 3.0 | - | | | 0 | 0 |
| 48 | 2.25 | - | 150 | 6.00 | 340 | 450/900 | 3.0 | - | | | 1 | 1 |
| 49 | 2.25 | 5.0 | 150 | 6.00 | 340 | 450/900 | 3.0 | - | | | 1 | 1 |
| 50 | 2.25 | 10.0 | 150 | 6.00 | 340 | 450/900 | 3.0 | - | | | 1 | 1 |
| 51 | 2.25 | 20.0 | 150 | 6.00 | 340 | 450/900 | 3.0 | - | | | 1 | 1 |

*Operating conditions for the production of core-shell microparticles*

For the manufacturing of core-shell loaded-microparticles, aqueous alginate solutions (Russo et al., 2020) at different concentrations (1.50% - 2.25%, (w/v)) were used to form the shell of the gel beads and an (O/W/O) emulsion was used to form the core. A primary O/W emulsion was formed by 1 mL dichloromethane and a 15 mL $CaCl_2$ aqueous solution at different concentrations, between 13.4 g/L and 26.8 g/L, while different percentages of PVA (0.5% - 1.0%, (w/v)) were used as a surfactant in the aqueous phase. This primary emulsion was added to 30 mL sunflower oil containing 0.5-1.0% Span® 85 (v/v) under gentle magnetic stirring. For the production of drug-loaded microparticles, ketoprofen lysinate was added in the range e 1% - 25% (w/w) regarding the amount of polymer used. Alginate capsules were prepared using a prilling apparatus (Encapsulator B-360, Büchi Labortechnik AG, St. Gallen, Switzerland) and to manufacture uniform core-shell beads in a narrow size distribution, concentric nozzles of different diameters were tested: inner nozzle ranging from 300 to 450 µm and outer nozzle ranging to 600 to 900 µm. The alginate solutions flow rate was tested in the 47-150 bar range whereas the emulsion flow rate was tested in the 4-6 mL/min range. The vibration frequency was tested in the range of 280 Hz-400 Hz, the amplitude was 5, and the use of the electrode was in the range of 500-2500V. Different amounts of ketoprofen lysinate were dissolved into the aqueous phase of the O/W/O emulsion in order to obtain 5%, 10%, and 20% (w/w) drug-loaded emulsions. The obtained gel beads were collected in a 0.3M $CaCl_2$ aqueous or ethanolic solution placed at different distances (3-5 cm) from the nozzle exit and maintained under gentle stirring for 5 minutes before being rinsed and collected in water or absolute ethanol, respectively.
